# Long non-coding RNA NEAT1 mediates MPTP/MPP^+^-induced apoptosis via regulating the miR-124/KLF4 axis in Parkinson’s disease

**DOI:** 10.1515/biol-2020-0069

**Published:** 2020-09-06

**Authors:** Jiyao Liu, Defang Liu, Bo Zhao, Cunwei Jia, Yunli Lv, Jun Liao, Kai Li

**Affiliations:** Department of Neurology, Anning Branch of the 940th Hospital of Joint Logistic Support Force of PLA, No. 1026, East-Anning Road, Lanzhou, Gansu, China

**Keywords:** Parkinson’s disease, NEAT1, KLF4, miR-124, apoptosis

## Abstract

Accumulating evidence suggests that dysregulation of long non-coding RNAs is closely associated with various human diseases, including Parkinson’s disease (PD). However, the role of nuclear-enriched abundant transcript 1 (NEAT1) in the PD process remains unclear. The number of TH+ cells was reduced, and the expression levels of NEAT1 and Krüppel-like factor 4 (KLF4) were increased in the midbrain of MPTP-HCl-treated mice. In addition, the expression of cleaved-caspase-3 (cleaved-casp-3) and Bax (apoptosis-related proteins) was increased, while the expression of Bcl-2 (anti-apoptotic protein) was reduced in MPTP-HCl-treated mice. The expression levels of NEAT1 and KLF4 were increased in MPP^+^-treated SH-SY5Y cells. Knockdown of NEAT1 promoted cell viability and decreased apoptosis in MPP^+^-treated SH-SY5Y cells, which could be reversed by upregulating KLF4. KLF4 was verified as a direct target of miR-124, and miR-124 could particularly bind to NEAT1. Downregulation of NEAT1 significantly increased cell viability and decreased apoptosis by regulating miR-124 expression in MPP^+^-treated SH-SY5Y cells. Additionally, interference of NEAT1 increased the number of TH+ cells and miR-124 expression, while reduced apoptosis and expression of KLF4 *in vivo*. NEAT1 knockdown increased cell viability and suppressed apoptosis in PD via regulating the miR-124/KLF4 axis, providing a promising avenue for the treatment of PD.

## 1 Introduction

Parkinson’s disease (PD) is the second most common multifactorial neurodegenerative disease, and it is estimated to affect 7–10 million people worldwide, especially older adults [1]. At the same time, statistics show that the incidence of PD continues to increase, and the trend is relatively young [2,3]. PD is caused by the loss of dopaminergic neurons in the substantia nigra, striatum and cerebral cortex. The main symptoms include bradykinesia, stiffness, resting tremor and unstable posture [4,5]. Due to complex causes and mechanisms, the current treatments, including drugs and surgery, can only reduce the symptoms of PD but cannot inhibit the development of PD. Therefore, it is imperative to explain the pathologic mechanisms at the molecular level in order to provide a potentially effective treatment strategy for PD.

Long non-coding RNAs (lncRNAs) are defined as a type of transcript with more than 200 nucleotides and without protein-coding ability [6]. LncRNAs have been reported to be involved in various cellular and physiological processes [7]. Nuclear-enriched abundant transcript 1 (NEAT1) has been identified as two isoforms (3.7 kb NEAT1-1 and 23 kb NEAT1-2), and it acts as an oncogene in many cancers [8,9]. Some studies have shown that NEAT1 plays an important role in degenerative disorders of the central nervous system, such as Huntington disease and amyotrophic lateral sclerosis [10,11]. More importantly, previous studies indicated that NEAT1 was overexpressed in PD and could regulate its progression [12]. However, detailed functions of NEAT1 in PD and its potential molecular mechanism remain poorly understood.

Krüppel-like factor 4 (KLF4), a member of the Krüppel-like family of zinc-finger transcription factors, was reported to be involved in multiple pathophysiological processes including cell growth, proliferation, differentiation and embryogenesis [13]. KLF4 has been reported to function as a tumor suppressor or as an oncogene in different cancers [14]. Moreover, it has been reported that KLF4 overexpression contributes to MPP^+^-induced neurotoxicity in M17 cells [15]. However, the connection between NEAT1 and KLF4 has not been elucidated, and more functions of KLF4 in PD have not been well clarified.

In recent years, the competing endogenous RNA (ceRNA) hypothesis has suggested that many lncRNAs may act as molecular sponges of microRNAs (miRNAs) to influence target messenger RNA (mRNA) expression, indicating the importance of this interaction during the disease process [16,17]. A previous study has demonstrated that miRNAs are widely involved in nervous system growth regulation and have a part to play in PD [18]. Recently, studies have shown that miR-124 is highly expressed in the brain compared to other organs and downregulated in the 1-methyl-4-phenyl-1,2,3,6-tetrahydropyridine (MPTP)-treated mouse model of PD [19,20]. Bioinformatics analysis exhibits the putative binding sites between miR-124 and NEAT1 or KLF4. Hence, we hypothesized that NEAT1 might regulate the progression of PD by acting as a ceRNA of miR-124 to modulate KLF4 expression.

In this study, we detected the number of TH+ cells and the expression levels of NEAT1 and KLF4 in the brain of MPTP-treated mice. Moreover, we explored the effects of NEAT1 and KLF4 on cell viability and apoptosis in MPP^+^-treated SH-SY5Y cells and investigated the ceRNA regulatory network of NEAT1/miR-124/KLF4. The aim of this study is to explain PD pathogenesis and provide a theoretical basis for the treatment of PD.

## 2 Materials and methods

### 2.1 Animals and treatment

Six-week-old female C57BL/6 mice were obtained from the Henan Experimental Animals Centre (Zhengzhou, China). A total of 14 mice were randomly divided into two groups (*n* = 7 per group). MPTP is a neurotoxin and has been widely used to establish PD animal models in many studies. To establish the mouse model of PD, one group received intraperitoneal injection of MPTP–HCl (30 mg/kg free base; Sigma-Aldrich, St Louis, MO, USA) per day. The control group received only the same volume of sterile saline solution (0.9%). Seven days after injection, all mice were sacrificed to remove the midbrains. Subsequently, the midbrains were stored at −80°C until experiments were carried out.

To explore the role of NEAT1 in PD mice, 2 days before the establishment of the PD mouse model, lentivirus-mediated short hairpin RNA (shRNA) targeting NEAT1 (sh-NEAT1) or its negative control (sh-NC) established by GeneCopoeia (Rockville, MD, USA) was injected into the midbrain of mice. Subsequently, the mice were treated as described above.


**Ethical approval:** The research related to animal use has been complied with all the relevant national regulations and institutional policies for the care and use of animals, and has been approved by the ethics committee of Anning Branch of the 940th Hospital of Joint Logistic Support Force of PLA. All operations were carried out in accordance with the Guide for the Care and Use of Laboratory Animals [21].

### 2.2 Cell culture and transfection

Human neuroblastoma SH-SY5Y cells were purchased from the American Type Culture Collection (ATCC; Rockville, MD, USA). The cells were cultured in DMEM (Hyclone, Logan, Utah, USA) supplemented with 10% fetal bovine serum (Gibco, Carlsbad, CA, USA) at 37°C in an incubator with 5% CO_2_. For MPP^+^ treatment, SH-SY5Y cells were treated with 0.5, 1 or 2 mM MPP^+^ (Sigma-Aldrich) for 24 h.

ShRNA targeting NEAT1 (sh-NEAT1) and its negative control (sh-NC) were constructed by GenePharma (Shanghai, China). The KLF4 expression plasmid (pcDNA-KLF4), its negative control (pcDNA-control), miR-124 mimic (miR-124) and its negative control (miR-NC) were purchased from Hanbio Biotechnology Co., Ltd (Shanghai, China). SH-SY5Y cells were transfected with oligonucleotides or vectors using Lipofectamine 3000 (Invitrogen, Carlsbad, CA, USA) according to the manufacturer’s instructions.

### 2.3 TH+ neurons

The midbrain sections that underwent cryostat-cut were incubated with a rabbit polyclonal anti-TH antibody (1:1,000; Cell Signaling Technology, Beverly, MA, USA) at 4°C overnight and then treated with biotinylated goat anti-rabbit IgG (1:2,000; Cell Signaling Technology) for 2 h. TH+ cells were counted using stereo investigator software.

### 2.4 Quantitative reverse transcription-polymerase chain reaction (qRT-PCR) assay

Total RNA was isolated from midbrain tissues or SH-SY5Y cells using Trizol reagent (Invitrogen). Complementary DNA (cDNA) was synthesized using a TaqMan Reverse Transcription Kit or TaqMan microRNA Reverse Transcription Kit (Applied Biosystems, Foster City, CA, USA). After reverse transcription, real-time PCR was carried out using a standard SYBR Green PCR kit (Thermo Fisher Scientific) on a CFX96 real-time PCR system (Bio-Rad, Hercules, CA, USA) according to the amplification instructions. All primers were purchased from Sangon Biotech (Shanghai, China), and the primer sequences were as follows: NEAT1 (forward 5′-CTTCCTCCCTTTAACTTATCCATTCAC-3′, reverse 5′-CTCTTCCTCCACCATTACCAACAATAC-3′), KLF4 (forward 5′-GAAATTCGCCCGCTCCGATGA-3′, reverse 5′-CTGTGTGTTTGCGGTAGTGCC-3′), miR-124 (forward 5′-GCTAAGGCACGCGGTG-3′, reverse 5′-GTGCAGGGTCCGAGGT-3′), GAPDH (forward 5′-TATGATGATATCAAGAGGGTAGT-3′, reverse 5′-TGTATCCAAACTCATTGTCATAC-3′), U6 (forward 5′-CTCGCTTCGGCAGCACATATACT-3′), reverse 5′-ACGCTTCACGAATTTGCGTGTC-3′). The relative expression levels of NEAT1, KLF4 and miR-124 were calculated using the 2^−ΔΔCt^ method and normalized to GAPDH or U6 serving as internal controls.

### 2.5 Western blot assay

The tissues and SH-SY5Y cells were lysed using RIPA lysis buffer (Sigma-Aldrich) containing a protease inhibitor (Sigma-Aldrich) for 20 min on ice. Subsequently, the cells were centrifuged at 12,000 rpm for 15 min to collect the protein supernatant. The concentration of protein was measured using a BCA protein assay kit (Beyotime, Shanghai, China). Total protein (30 µg) was separated by 10–12% sodium dodecyl sulfate-polyacrylamide gel electrophoresis and then transferred onto a polyvinylidene fluoride membrane (Millipore Corp., Atlanta, GA, USA). Next, the membranes were blocked using Tris-buffered saline Tween supplemented with 5% non-fat milk for 2 h at room temperature. The membranes were then incubated with primary antibodies against cleaved-caspase-3 (cleaved-casp-3) (1:1,000; #9664), Bax (1:1,000; #14796), Bcl-2 (1:1,000; #3498), KLF4 (1:1,000; #12173) and GAPDH (1:1,000; #5174) (Cell Signaling Technology, Beverly, MA, USA) overnight at 4°C, followed by incubation with horseradish peroxidase-conjugated goat anti-rabbit IgG (Sangon Biotech) at 1:4,000 dilution for 2 h. Finally, the membranes were examined with enhanced chemiluminescence (Tanon, Shanghai, China) and imaged using a chemiluminescence gel imaging system (Tanon). Protein levels were quantified using ImageJ software and normalized to GAPDH.

### 2.6 MTT assay

Cell viability was detected using a 3-(4,5-dimethylthiazol-2-yl)-2,5-diphenyl tetrazolium bromide assay. Briefly, SH-SY5Y cells were seeded in 96-well plates at a density of 2 × 10^4^ cells/mL and cultured overnight. Then, SH-SY5Y cells were treated with MPP^+^ (2 mM) for 24 h and transfected with sh-NC, sh-NEAT1, sh-NEAT1 + pcDNA-control, sh-NEAT1 + pcDNA-KLF4, sh-NEAT1 + miR-NC or sh-NEAT1 + miR-124. After transfection for 48 h, 20 µL of MTT solution (5 mg/mL) was added to each well. After further incubation for 4 h, the culture medium mixture was discarded, and the formazan crystals were dissolved by adding 150 µL of dimethyl sulfoxide (Sigma-Aldrich) to each well. Cell viability was assessed at 490 nm using a microplate reader (Bio-Rad, Hercules, CA, USA).

### 2.7 Apoptosis assay

The apoptosis rate was measured using flow cytometry with an Annexin V-fluorescein isothiocyanate (FITC)/propidium iodide (PI) apoptosis detection kit (Sangon Biotech). SH-SY5Y cells were seeded in six-well plates and treated with MPP^+^ (2 mM) for 24 h and transfected with different transfection reagents. After 48 h, the cells were collected and stained with Annexin V-FITC and PI in a dark environment for 15 min. The percentage of apoptotic cells was examined using flow cytometry (BD Biosciences, Franklin Lakes, NJ, USA).

### 2.8 Luciferase reporter assay

The putative binding sites of miR-124 and NEAT1 or KLF4 were predicted using online software MiRcode Tool or TargetScan Tool. Partial fragments of wild-type NEAT1 (WT-NEAT1), mutant NEAT1 (MUT-NEAT1), 3′-untranslated regions of KLF4 wild type (KLF4 3′-UTR-WT) or 3′-untranslated regions of KLF4 mutant (KLF4 3′-UTR-MUT) were amplified and cloned into pGL3 plasmids (Promega, Madison, WI, USA). The reporter vectors with miR-NC, miR-124, miR-124 + pcDNA-control or miR-124 + pcDNA-NEAT1 were co-transfected into SH-SY5Y cells according to the manufacturer’s protocols. Luciferase activities were detected with a dual luciferase reporter assay kit (Promega) after transfection for 48 h.

### 2.9 Statistical analysis

In the present study, all data from at least three individual experiments were expressed as mean ± standard deviation. Differences between the two groups were analyzed using GraphPad Prism 5.0 software (GraphPad Prism, San Diego, CA). A *P* value of <0.05 was considered statistically significant.

## 3 Results

### 3.1 Effects of MPTP and MPP^+^ on NEAT1 and KLF4 expression *in vivo* and *in vitro*

**Figure 1:**
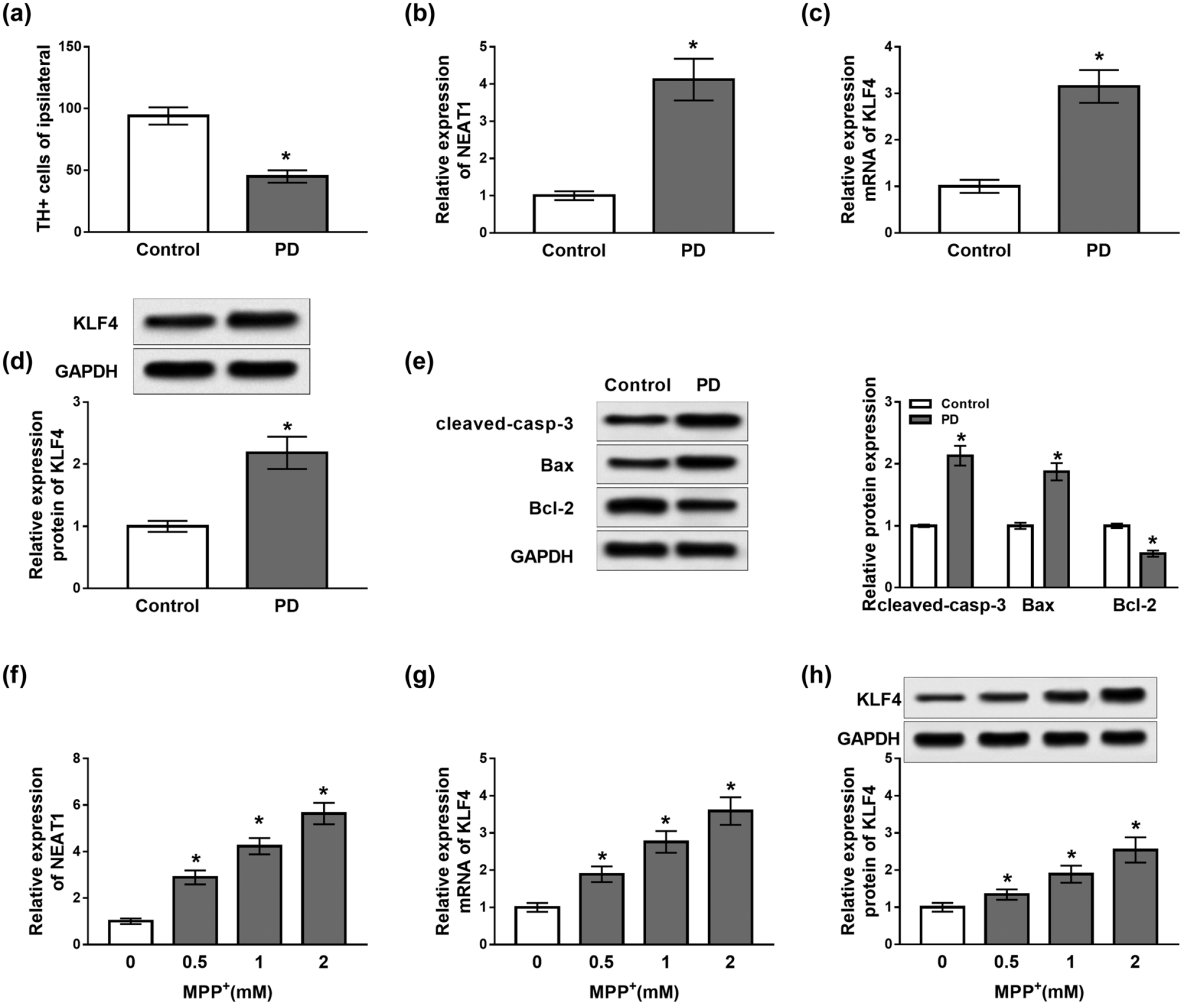
Effects of MPTP on NEAT1 and KLF4 expression *in vivo* and *in vitro*. (a) The number of TH+ neurons was dramatically decreased in PD mice. (b) Abundance of NEAT1 detected in the midbrain of PD mice by qRT-PCR. (c and d) mRNA and protein levels of KLF4 detected by qRT-PCR and western blot, respectively. (e) Expression of apoptosis-related proteins analyzed by western blot. (f) Expression of NEAT1 measured in SH-SY5Y cells treated with MPP+. (g and h) mRNA and protein levels of KLF4 examined in MPP^+^-treated SH-SY5Y cells. *P < 0.05.

In order to detect the number of TH+ cells and the expression of NEAT1 and KLF4 in the brain of MPTP-treated mice (*n* = 7), MPTP–HCl was injected into a group of mice, and a sterile saline solution (0.9%) was injected into the control group. The number of TH+ cells was dramatically reduced in PD mice in contrast to the control group (*P* < 0.05, Figure 1a). However, the results of qRT-PCR showed that NEAT1 and KLF4 expression levels were upregulated in the midbrain of PD mice (*P* < 0.05, Figure 1b and c). Besides, KLF4 protein abundance was also increased in the midbrain of PD mice (*P* < 0.05, Figure 1d). In addition, the expression of apoptosis-related proteins was analyzed by western blot. Results showed that the levels of cleaved-casp-3 and Bax proteins were significantly elevated in the midbrain of PD mice, while the expression of Bcl-2 protein was significantly decreased (*P* < 0.05, Figure 1e). Furthermore, to explore the effects of MPP^+^ on neuronal cells, human SH-SY5Y cells were treated with different concentrations (0, 0.5, 1 and 2 mM) of MPP^+^ for 24 h, and the cells without MPP^+^ treatment were used for the control group. The results revealed that the expression levels of NEAT1 and KLF4 were increased in a dose-dependent manner in SH-SY5Y cells treated with MPP^+^ (*P* < 0.05, Figure 1f and g). Similarly, expression of KLF4 protein was also enhanced in a dose-dependent manner (*P* < 0.05, Figure 1h). These results indicated that NEAT1 and KLF4 might play vital roles in the development of PD.

### 3.2 Effects of NEAT1 on cell viability and apoptosis in MPP^+^-treated SH-SY5Y cells

**Figure 2:**
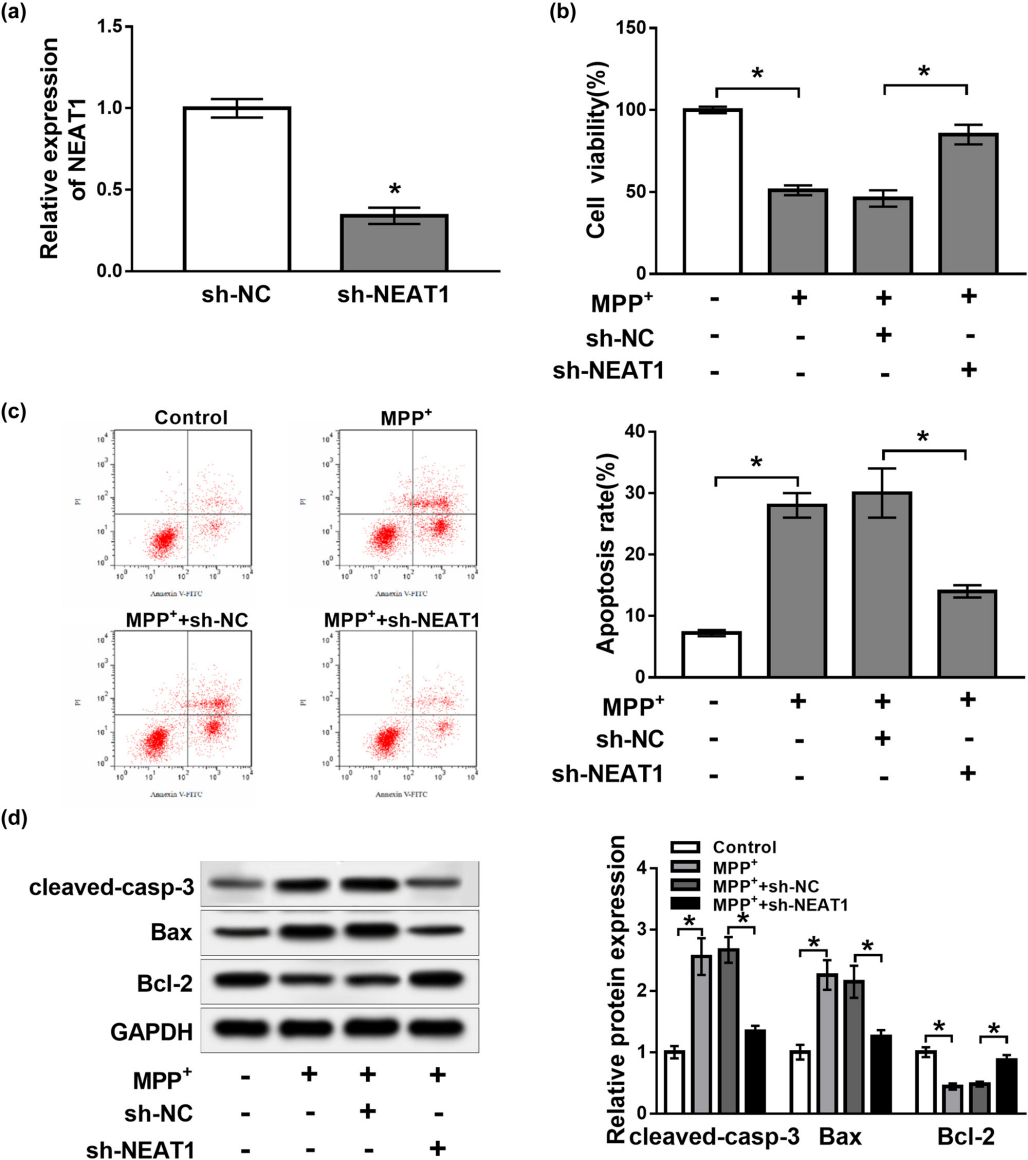
Knockdown of NEAT1 increased cell viability and reduced apoptosis in SH-SY5Y cells treated with MPP^+^. (a) Expression of NEAT1 detected by qRT-PCR in SH-SY5Y cells transfected with sh-NC or sh-NEAT1. (b–d) SH-SY5Y cells were transfected with sh-NC or sh-NEAT1 and then treated with MPP^+^ (2 mM). (b and c) Cell viability and apoptosis analyzed by MTT assay or flow cytometry, respectively. (d) Expression of apoptosis-related proteins analyzed by western blot. *P < 0.05.

To investigate the effect of NEAT1 on PD progression, sh-NEAT1 or sh-NC was transfected into SH-SY5Y cells and then treated with MPP^+^ (2 mM). According to Figure 2a (*P* < 0.05), transfection of sh-NEAT1 led to an obvious reduction of NEAT1 expression in SH-SY5Y cells compared with the sh-NC group, suggesting that sh-NEAT1 was successfully transfected into SH-SY5Y cells. Cell viability and apoptosis were measured by MTT assay and flow cytometry analysis, respectively. Results showed that cell viability was evidently inhibited, while the apoptosis rate was conspicuously increased in SH-SY5Y cells treated with MPP^+^, which was reversed by the inhibition of NEAT1 (*P* < 0.05, Figure 2b and c). Western blot analysis displayed that cleaved-casp-3 and Bax protein levels were notably increased, while the Bcl-2 protein level was prominently suppressed in SH-SY5Y cells treated with MPP^+^, whereas these effects were reversed by transfection of sh-NEAT1 (*P* < 0.05, Figure 2d). Our data suggested that knockdown of NEAT1 elevated cell viability and restrained apoptosis in SH-SY5Y cells treated with MPP^+^.

### 3.3 KLF4 overexpression reversed the effects of NEAT1 knockdown on cell viability and apoptosis in MPP^+^-treated SH-SY5Y cells

**Figure 3:**
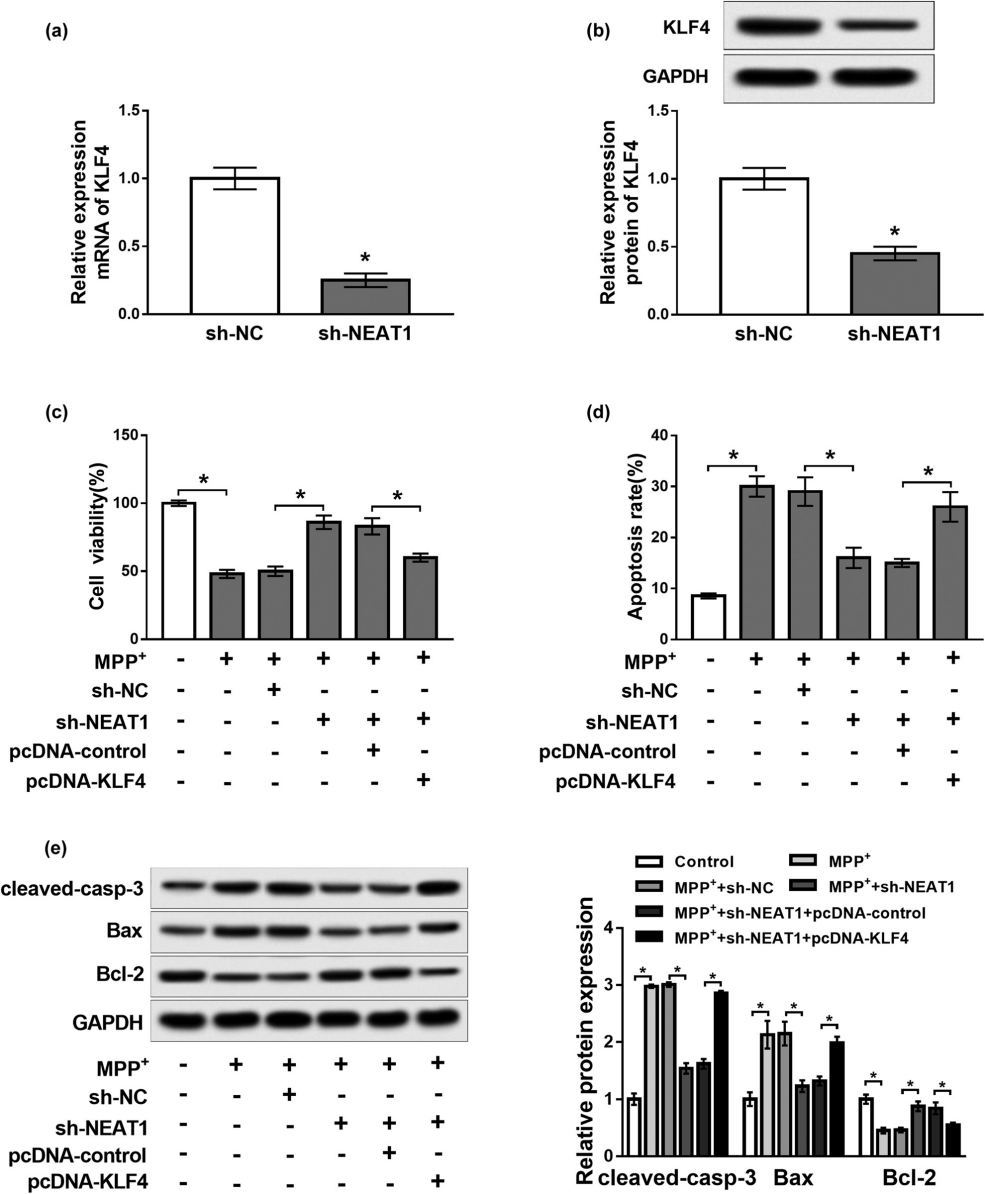
KLF4 overexpression reversed the effects of NEAT1 knockdown on cell viability and apoptosis in MPP^+^-treated SH-SY5Y cells. (a and b) mRNA and protein abundance of KLF4 analyzed in SH-SY5Y cells transfected with sh-NC or sh-NEAT1. (c–e) SH-SY5Y cells were transfected with sh-NC, sh-NEAT1, sh-NEAT1 + pcDNA-control or sh-NEAT1 + pcDNA-KLF4 and then exposed to MPP^+^. (c and d) Cell viability and apoptosis measured by MTT assay and flow cytometry, respectively. (e) Expression of apoptosis-related proteins analyzed by western blot. *P < 0.05.

To explore the effect of NEAT1 on PD and the relationship between KLF4 and NEAT1, SH-SY5Y cells were transfected with sh-NC, sh-NEAT1, sh-NEAT1 + pcDNA-control or sh-NEAT1 + pcDNA-KLF4 and then exposed to MPP^+^. qRT-PCR and western blot analysis revealed that knockdown of NEAT1 evidently suppressed the mRNA and protein levels of KLF4 compared with the control group (*P* < 0.05, Figure 3a and b). Upregulation of KLF4 abolished the effects of NEAT1 knockdown on promotion of cell viability and inhibition of the apoptosis rate in MPP^+^-treated SH-SY5Y cells (*P* < 0.05, Figure 3c and d). Moreover, western blot analysis revealed that overexpression of KLF4 reversed the effects of NEAT1 knockdown on the decrease of cleaved-casp-3 and Bax protein expression and the increase of Bcl-2 protein expression (*P* < 0.05, Figure 3e). These data demonstrated that overexpression of KLF4 reversed the effects of NEAT1 knockdown on cell viability and apoptosis in MPP^+^-treated SH-SY5Y cells.

### 3.4 NEAT1 targeted miR-124 to regulate KLF4 expression

**Figure 4:**
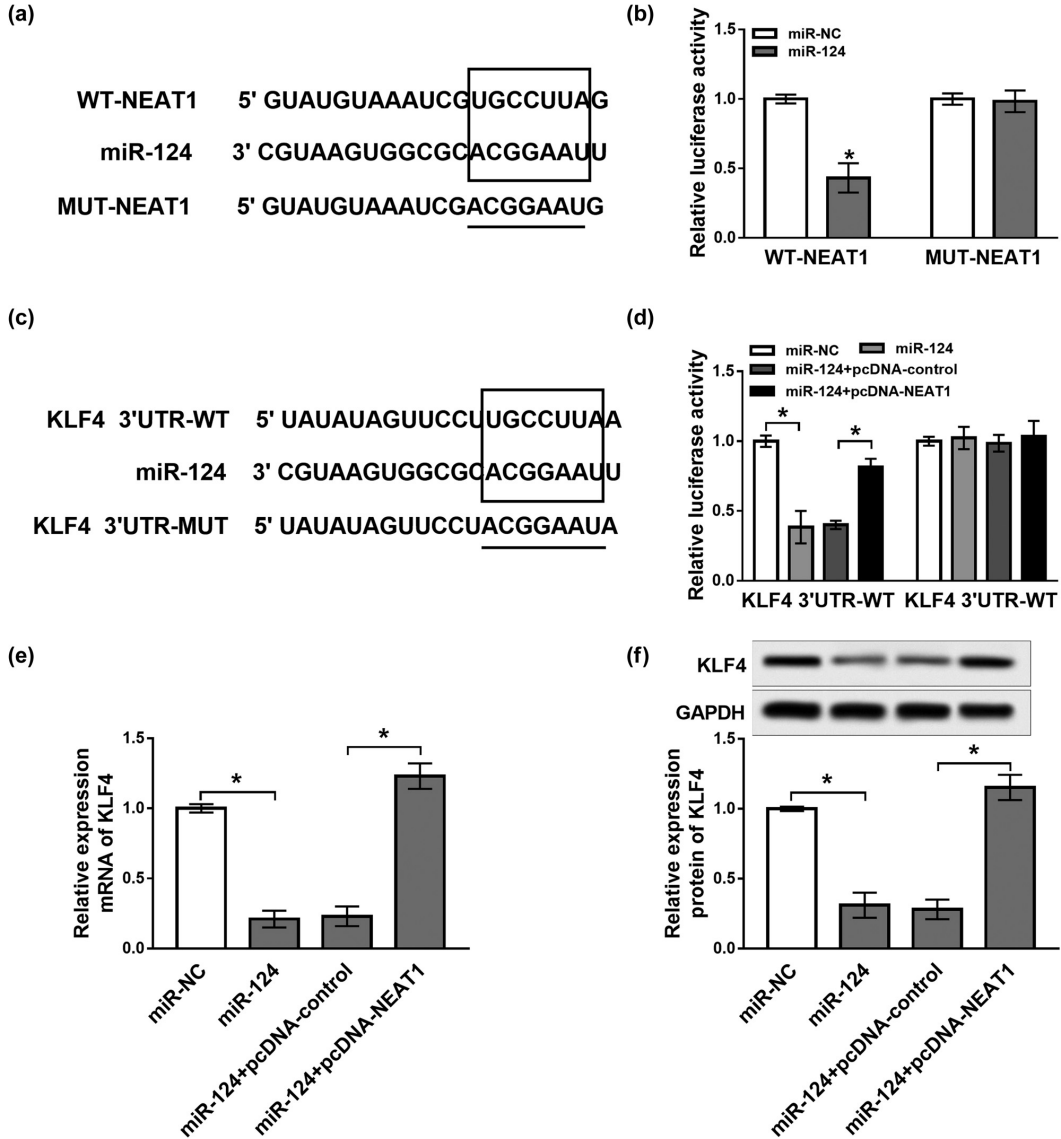
NEAT1 targeted miR-124 to regulate KLF4 expression. (a) The potential binding sites of NEAT1 and miR-124 predicted by MiRcode Tool. (b) Luciferase activity measured in SH-SY5Y cells co-transfected with WT-NEAT1 or MUT-NEAT1 and miR-124 or miR-NC by a dual luciferase reporter assay. (c) The putative binding sites of miR-124 and KLF4 predicted by TargetScan Tool. (d) Luciferase activity detected in SH-SY5Y cells co-transfected with different reagents. (e and f) mRNA and protein expression of KLF4 evaluated in SH-SY5Y cells transfected with miR-NC, miR-124, miR-124 + pcDNA-control or miR-124 + pcDNA-NEAT1. *P < 0.05.

The potential binding sites of NEAT1 and miR-124 were predicted by MiRcode Tool, and we found that miR-124 was a potential binding target of NEAT1 (*P* < 0.05, Figure 4a). Next, the prediction was confirmed by a dual luciferase reporter assay. Results showed that the transfection of miR-124 decreased the relative luciferase activity of the WT-NEAT1 reporter, whereas the effect was small with respect to the relative luciferase activity of the MUT-NEAT1 reporter (*P* < 0.05, Figure 4b). Subsequently, online software TargetScan Tool also showed that miR-124 might bind to KLF4 3′-UTR (*P* < 0.05, Figure 4c). Then, pGL3 luciferase reporter plasmids containing the 3′-UTR sequences of KLF4-WT and KLF4-MUT were established. We found that the luciferase activity of KLF4 3′-UTR-WT was reduced in SH-SY5Y cells transfected with miR-124, which was abrogated by the addition of pcDNA-NEAT1 (*P* < 0.05, Figure 4d). Similarly, the luciferase activity of KLF4 3′-UTR-MUT was unaffected by transfection of miR-124 and pcDNA-NEAT1. Moreover, qRT-PCR and western blot analysis indicated that overexpression of miR-124 markedly suppressed the mRNA and protein levels of KLF4 in contrast to the control group, which was abated by transfection of pcDNA-NEAT1 (*P* < 0.05, Figure 4e and f). Our preliminarily data suggest that NEAT1 regulated KLF4 expression by targeting miR-124.

### 3.5 NEAT1 could regulate cell viability and apoptosis in MPP^+^-treated SH-SY5Y cells by sponging miR-124

**Figure 5:**
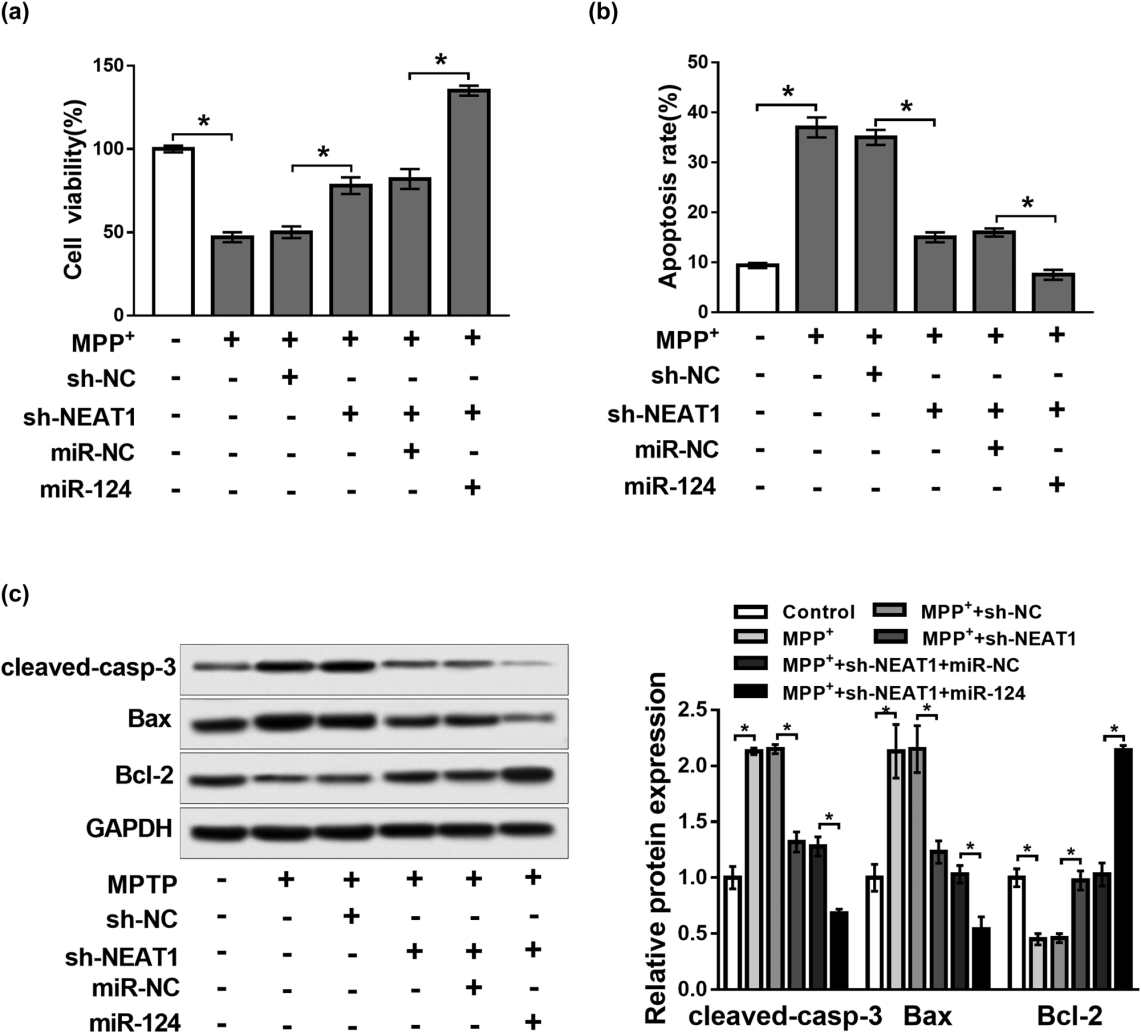
NEAT1 regulated cell viability and apoptosis in MPP^+^-treated SH-SY5Y cells by targeting miR-124. SH-SY5Y cells were transfected with sh-NC, sh-NEAT1, sh-NEAT1 + miR-NC or sh-NEAT1 + miR-124 and then treated with MPP^+^ (2 mM). (a) Cell viability assessed by MTT assay. (b) Cell apoptosis determined by flow cytometry. (c) Expression of apoptosis-related proteins detected by western blot. *P < 0.05.

To determine the regulatory network of NEAT1 and miR-124 in PD, SH-SY5Y cells were transfected with sh-NEAT1, sh-NC sh-NEAT1 + miR-124 or sh-NEAT1 + miR-NC and then treated with MPP^+^ (2 mM). As shown in Figure 5a and b (*P* < 0.05), transfection of sh-NEAT1 led to an obvious increase of cell viability and a decrease of apoptosis rate in MPP^+^-treated SH-SY5Y cells compared with the transfection of sh-NC, and these effects were improved by the addition of miR-124. Also, western blot analysis revealed that knockdown of NEAT1 resulted in a significant decrease in cleaved-casp-3 and Bax protein levels, while an increase in Bcl-2 protein expression in SH-SY5Y cells treated with MPP^+^ when compared with the sh-NC transfection group, and addition of miR-124 could enhance these effects (*P* < 0.05, Figure 5c). These findings suggest that NEAT1 targets miR-124 to regulate cell viability and apoptosis in SH-SY5Y cells treated with MPP^+^.

### 3.6 Effects of NEAT1 knockdown on the PD mouse model

**Figure 6:**
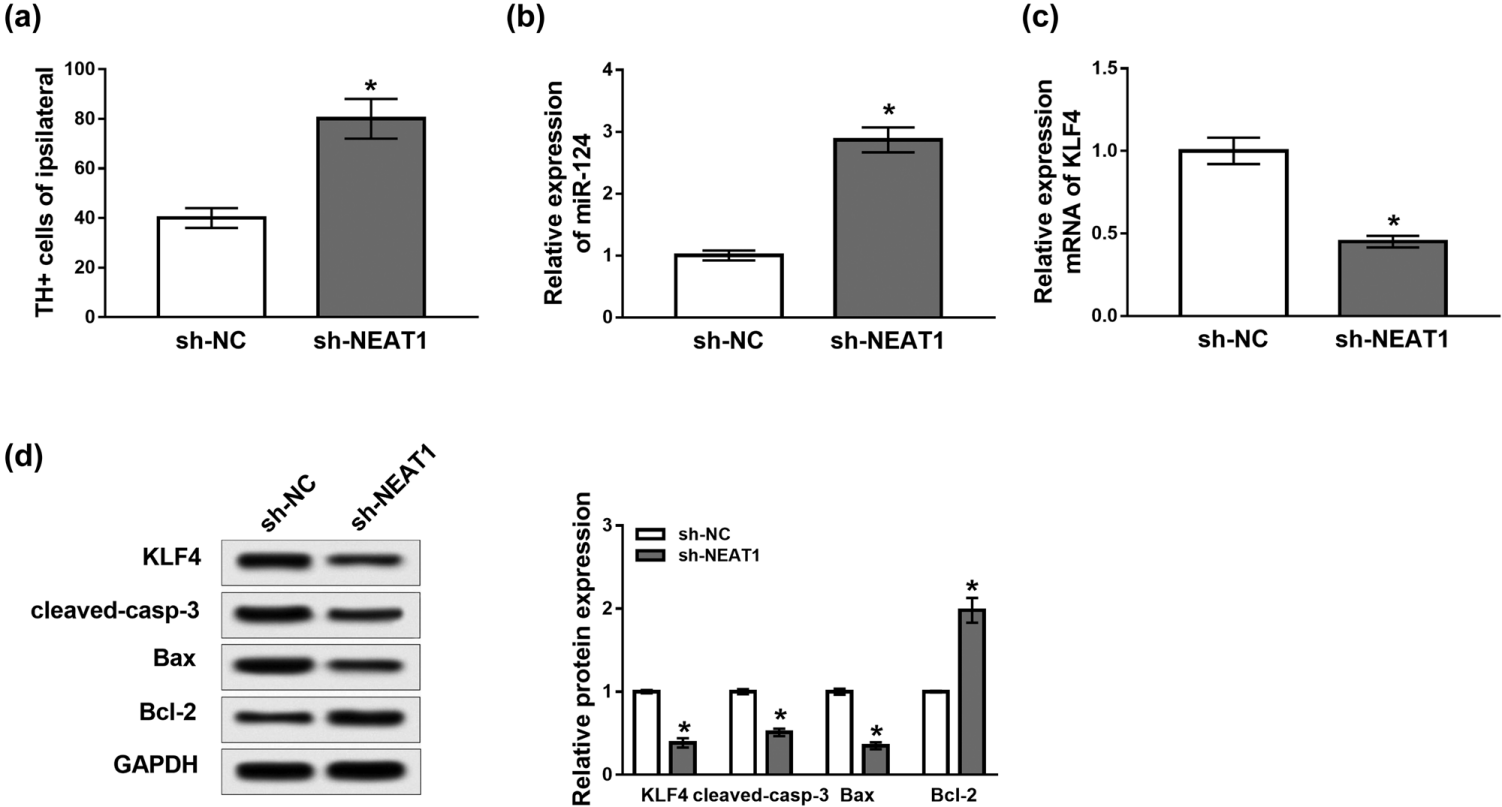
The role of NEAT1 knockdown in the PD mouse model. Lenti-sh-NC or lenti-sh-NEAT1 was injected into the midbrain of mice 2 days before the establishment of the PD mice. (a) The number of TH+ neurons in the midbrain of PD mice. (b and c) Expression levels of miR-124 and KLF4 measured by qRT-PCR in the midbrain of PD mice. (d) The levels of KLF4, cleaved-casp-3, Bax and Bcl-2 proteins analyzed by western blot. *P < 0.05.

To explore the effects of NEAT1 on PD *in vivo*, sh-NC or lenti-sh-NEAT1 was injected into the midbrain of mice 2 days before the establishment of the PD mouse model. Results showed that the number of TH+ neurons was obviously increased in PD mice transfected with lenti-sh-NEAT1 (*P* < 0.05, Figure 6a). Moreover, qRT-PCR revealed that the expression of miR-124 was significantly increased in PD mice transfected with lenti-sh-NEAT1, whereas the mRNA expression of KLF4 was notably decreased (*P* < 0.05, Figure 6b and c). In addition, injection of lenti-sh-NEAT1 clearly reduced the levels of KLF4, cleaved-casp-3 and Bax proteins while elevating the expression of Bcl-2 protein (*P* < 0.05, Figure 6d). These results suggested that knockdown of NEAT1 might inhibit the development of PD *in vivo*.

## 4 Discussion

PD is the second most common neurodegenerative disorder, ranking only behind Alzheimer’s disease [22]. At present, the establishment of MPTP-induced PD models has greatly improved our understanding of PD pathogenesis and provided a possible avenue to discover new therapies [23]. Recently, it has been revealed that lncRNAs are particularly abundant in the nervous system and play important roles in many neurobiological and neurodegenerative diseases [24,25]. In addition, lncRNA can be used as a precise biomarker for PD and other neurodegenerative diseases [26]. In this study, MPTP and MPP^+^ were used to establish PD models *in vivo* and *in vitro*, respectively, and we focused on the role and relationship of NEAT1, miR-124 and KLF4 in PD.

NEAT1, a highly abundant ncRNA, has been suggested to be involved in cellular differentiation and stress response through the paraspeckle pathway [8]. It has been reported that NEAT1 was upregulated in the PD mouse model, and downregulation of NEAT1 could effectively suppress MPTP-induced apoptosis and autophagy in PD [12,27]. Consistent with these studies, NEAT1 was upregulated in the midbrain of PD mice and in SH-SY5Y cells treated with MPP^+^. In addition, knockdown of NEAT1 elevated cell viability. Bax (pro-apoptotic) and Bcl-2 (anti-apoptotic) are the primary regulatory molecules in the Bcl-2 family and play important roles in apoptotic cells [28]. As a member of the caspase family, caspase-3 is a key executive molecule that regulates apoptosis, and the activated effector caspase-3 causes irreversible cell death [29,30]. In this study, cleaved-casp-3 and Bax protein levels were notably increased, while the Bcl-2 protein level was prominently suppressed in MPP^+^-treated SH-SY5Y cells. These results indicate that NEAT1 might contribute to the development of PD.

KLF4 is one of the first genes found in the KLF family, and it is widely detected in a variety of human tissues and plays essential roles in different physiological processes, including PD [31,32]. For example, KLF4 knockdown decreased apoptosis in MPP^+^-treated SH-SY5Y cells, while apoptosis was increased by upregulating KLF4 [33]. In addition, further research demonstrated that interference of KLF4 could protect SH-SY5Y against MPP^+^-induced cell apoptosis, and overexpression of KLF4 significantly weakened the protective effect of miR-7 on MPP^+^-induced apoptosis in SH-SY5Y cells [34]. Here, we found that KLF4 expression was markedly upregulated in the midbrain of PD mice and MPP^+^-treated SH-SY5Y cells. Furthermore, overexpression of KLF4 reversed the effects of NEAT1 knockdown on cell viability and apoptosis in MPP^+^-treated SH-SY5Y cells. These data suggest that NEAT1 knockdown inhibited the development of PD by downregulating KLF4.

Mounting evidence indicates that lncRNAs can function as miRNA sponges or decoys to compete for miRNA binding to protein-coding transcripts [35]. Here, we hypothesized that NEAT1 might act as a miRNA sponge to regulate PD progression. Bioinformatics analysis was preformed, and the results indicated that NEAT1 contained binding sites for miR-124. MiR-124 plays a key role in neuronal biology and is highly expressed in neurons [36]. Recently, several studies showed that miR-124 regulated apoptosis and autophagy in PD. For instance, miR-124 could regulate apoptosis and autophagy by regulating the AMPK/mTOR pathway and act as a protective agent for dopaminergic neurons during PD [37]. In addition, MALAT1 promoted cell apoptosis by targeting miR-124 in PD [38]. A previous study revealed that miR-124 could suppress the progression of hepatocarcinoma via targeting KLF4 [39]. Furthermore, upregulation of NEAT1 promoted the development and progression of nasopharyngeal carcinoma by regulating the miR-124/NF-κB signaling pathway [40]. However, there is no evidence to support the regulatory network NEAT1/miR-124/KLF4 in PD, and the underlying mechanism needs to be clarified. In our study, we found that miR-124 could directly bind to NEAT1 and KLF4, and NEAT1 functioned as a ceRNA via sponging miR-124 to regulate KLF4 expression in SH-SY5Y cells. Further studies revealed that NEAT1 could regulate cell viability and apoptosis in MPP^+^-treated SH-SY5Y cells by sponging miR-124, and the expression of miR-124 was significantly increased in PD mice transfected with lenti-sh-NEAT1. These findings proved that NEAT1 positively regulated KLF4 expression and negatively regulated miR-124 expression. Hence, it was speculated that NEAT1 exerted its functions by regulating the miR-124/KLF4 axis.

In conclusion, the number of TH+ cells was dramatically reduced and the expressions of NEAT1 and KLF4 were upregulated in the midbrain of mice injected with MPTP-HCl. In addition, knockdown of NEAT1 promoted cell viability and reduced apoptosis in MPP^+^-treated SH-SY5Y cells, which could be reversed by overexpression of KLF4. Moreover, NEAT1 acted as a molecular sponge of miR-124 to regulate KLF4 expression. Collectively, NEAT1 knockdown attenuated MPTP/MPP^+^-induced apoptosis via regulating the miR-124/KLF4 axis in PD, providing a novel mechanism for understanding PD progression.
